# The Vaginal Microbiota, Human Papillomavirus, and Cervical Dysplasia—A Review

**DOI:** 10.3390/medicina61050847

**Published:** 2025-05-05

**Authors:** Justė Kazlauskaitė, Guoda Žukienė, Vilius Rudaitis, Daiva Bartkevičienė

**Affiliations:** 1 Faculty of Medicine, Vilnius University, LT-03101 Vilnius, Lithuania; 2 Clinic of Obstetrics and Gynaecology, Faculty of Medicine, Institute of Clinical Medicine, Vilnius University, LT-03101 Vilnius, Lithuania; vilius.rudaitis@santa.lt (V.R.); daiva.bartkeviciene@mf.vu.lt (D.B.)

**Keywords:** vaginal microbiota, human papillomavirus infection, cervical precancerous lesions, cervical neoplasia

## Abstract

*Background and Objectives:* The relationship between the vaginal microbiota, human papillomavirus infection (HPV), and cervical precancerous lesions is a critical area of research, as it influences both the progression of HPV-related diseases and potential treatment strategies. New evidence suggests that *Lactobacillus crispatus* dominance in the microbiota may protect against HPV persistence and speed the elimination of HPV. This study aims to explore the relationship between the vaginal microbiota composition and HPV infection, focusing on the impact of these factors on the development of cervical precancerous lesions. *Materials and Methods:* A comprehensive literature review was conducted using the PubMed database, focusing on studies that analyzed the association between the vaginal microbiota and HPV infection in the context of cervical dysplasia. This study was primarily based on clinical data on HPV integration in women with low-grade squamous intraepithelial lesions (LSILs), high-grade squamous intraepithelial lesions (HSILs), and cervical cancer. *Results:* Different types of vaginal microbiota communities (CSTs) have different pathogenic or protective potential. Healthy women predominantly exhibited CST I, with *Lactobacillus crispatus* as the dominant microorganism. CST IV, associated with increased anaerobic bacteria, was most common in HSIL and cervical cancer patients. Statistical analysis revealed that bacterial vaginosis (BV) was significantly associated with HPV persistence, with studies reporting a 1.8–3.4-fold increased risk (*p* < 0.05) of persistent HR-HPV infection in BV-positive women. *Conclusions:* Our literature review suggests that the composition of the vaginal microbiota can modulate the local immune response, the expression of viral oncogenes, and the integrity of the epithelial barrier. Furthermore, certain bacterial genes or metabolic pathways can be associated with a favorable or unfavorable outcome of the disease. Analysis of the vaginal microbiota could serve as an additional risk assessment tool, helping to distinguish between regressing and progressive precancerous conditions.

## 1. Introduction

HPV is a common sexually transmitted virus [1]. However, only a small proportion of high-risk HPV (HR-HPV) infections progress to cervical precancerous lesions and cancer. Persistent HPV infection is needed for the development of cervical precancerous lesions [2]. The peak prevalence of infection is between the ages of 15 and 26 and around 45 years. It has been shown that the persistence of HPV infection increases with age, and its incidence decreases with age. According to the literature, HPVs include a group of more than 200 related viruses. At present, the HR-HPV types are identified as 16, 18, 31, 33, 35, 39, 45, 51, 52, 56, 58, 59, 68, 69, 73, and 82 [3,4,5,6].

Although HPV infection plays the key role in the development of cervical precancerous and cancerous lesions, the mechanisms involved in HPV infection pathophysiology suggest that HPV infection alone is insufficient for these changes to develop and that other factors are involved [7]. The mechanisms involved in the clearance or persistence of HPV seem to be related to the microbiota but are poorly understood. Still, with the development of research methods and technologies, special attention has been paid to the composition of the vaginal microbiota [8]. Studies suggest that microbial communities may influence the persistence of HPV infection and its progression to cancer through several mechanisms. These include modulating genital inflammation and immune responses, affecting the expression of high-risk HPV oncogenes and oncoprotein production, and regulating oxidative stress and DNA damage [9]. Although the vaginal microbiota is composed of a diverse range of microorganisms, *Lactobacillus* species dominate the vaginal microbiota of most healthy women [10]. Studies suggest that genital tract dysbiosis and certain bacteria may promote the progression of HPV infection and the development of cervical intraepithelial neoplasia (CIN). Decreased *Lactobacillus* species are associated with a higher risk of CIN and slower disease regression, creating an inflammatory environment that promotes expression of HPV oncogenes E6 and E7 (notably E6 degrades the p53 protein, and E7 inactivates the retinoblastoma protein pRb) [11].

The need for a deeper investigation into this topic arises from several important perspectives. From an epidemiological standpoint, cervical cancer prevention remains a global public health priority, particularly in regions where access to HPV vaccination and cytological screening is limited [12]. The precise mechanisms by which the vaginal microbiome influences HPV persistence and neoplastic progression are not yet fully elucidated [13]. Recent metagenomic analyses have identified novel microbial pathways—particularly those related to lipid metabolism, amino acid biosynthesis, and modulation of the immune response—that may contribute to the persistence of HPV and the progression of cervical neoplastic lesions [14]. An improved understanding of microbiota–HPV interactions could lead to the development of innovative prevention and treatment strategies, including microbiome-based interventions such as probiotics or vaginal microbiota modulation therapies [12,15].

This article aims to present a comprehensive and current overview of recent scientific publications exploring the relationship between the vaginal microbiome, human papillomavirus (HPV) infection, and the onset of cervical precancerous lesions. Special emphasis is placed on insights gained through metagenomic research and the influence of ethnic variability in microbiome composition and HPV-related disease risk, highlighting the importance of developing personalized approaches to cervical cancer prevention.

## 2. Materials and Methods

### 2.1. Search Strategy

A literature review was conducted in the PubMed database, analyzing scientific articles in English from the past 10 years. Studies examining the impact of vaginal microbiota composition on the persistence of human papillomavirus infection, cervical dysplasia, and cancer progression were selected. The search combined the following terms using appropriate Boolean operators (i.e., AND, OR): “Vaginal microbiota”, “Human papillomavirus infection”, “Cervical precancerous lesions”. The same queries were applied across all databases.

### 2.2. Inclusion and Exclusion Criteria

Studies meeting the following criteria were included in the review:

1. Analysis of the composition of the vaginal microbiota assessed by molecular methods (next-generation sequencing);
2. Groups of reproductive-age and older women with different vaginal microbiota composition, women without HPV infection (healthy) and with HPV infection or cervical intraepithelial neoplasia.

Studies were excluded if they met any of the following criteria:

1. Conference papers or editorials;
2. Duplicated studies.

### 2.3. Data Analysis

The studies’ results and graphical representation were evaluated using Microsoft Excel (Microsoft Corp., Redmond, WA, USA).

### 2.4. Study Selection

**Figure 1:**
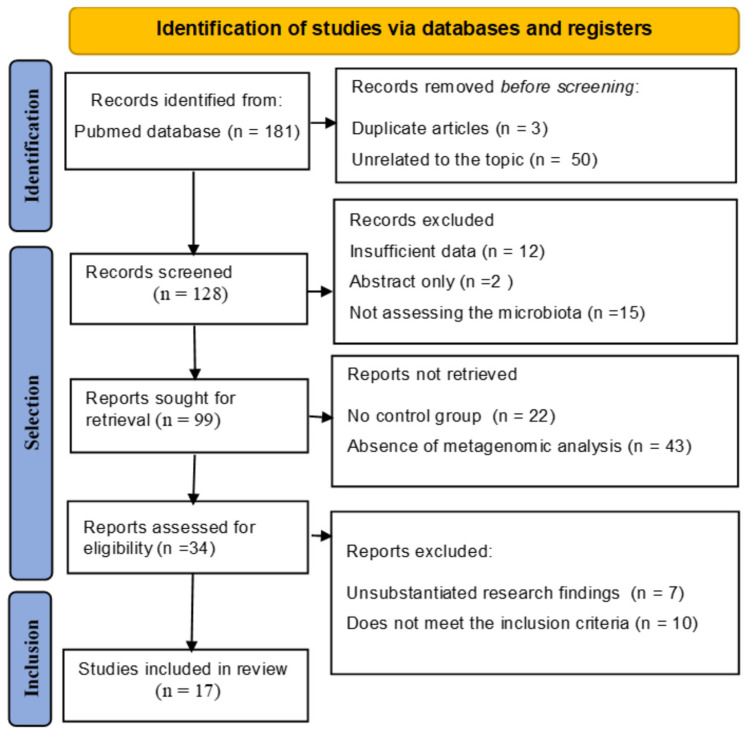
PRISMA Flow Diagram of Study Selection Process. This figure illustrates the PRISMA (Preferred Reporting Items for Systematic Reviews and Meta-Analyses) flow chart detailing the systematic review process for assessing the relationship between cervical microbiota, HPV, and cervical cancer progression.

A total of 181 articles were identified. After removing duplicates and those not meeting the inclusion criteria, a portion was selected for full-text review; 17 studies were determined to meet the inclusion criteria. The results are outlined in the PRISMA diagram (Figure 1).

## 3. Results

### 3.1. HPV Prevalence and Integration Mechanism

HPV is transmitted through skin-to-mucous contact, most commonly during sexual intercourse, and rarely vertically during childbirth. HPV types are divided into high-risk (e.g., HR-HPV 16, HR-HPV 18), which are associated with 70% of cervical cancer, and low-risk (e.g., HPV 6, HPV 11), which can cause genital warts [6,16,17]. The global prevalence of HPV is 11.7%, with the highest prevalence in Africa, Eastern Europe, and the Caribbean [16,17,18]. HR-HPV contributes to 99% of cervical precancerous lesions [19,20]. HPV infection occurs when virus particles enter basal cells, usually through epithelial microabrasions. The most vulnerable sites are the transitional epithelium of the cervix and the anus [21].

**Figure 2:**
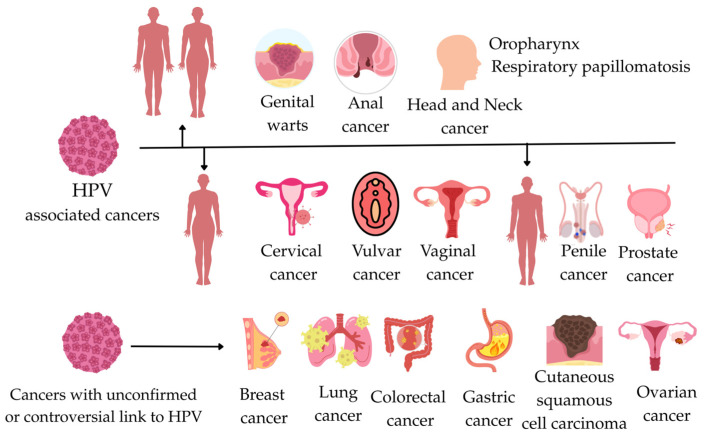
This figure illustrates the relationships between HPV-associated cancer and cancers with an unconfirmed or controversial link to HPV.

HPV is recognized as the primary etiological agent in virtually all cases of cervical cancer. Moreover, its oncogenic potential extends beyond the cervix, contributing to a significant proportion of oropharyngeal cancers and malignancies of the anogenital tract, including vaginal, vulvar, penile, and anal cancers [6,16,17]. HPV is responsible for approximately 690,000 cancer cases worldwide. Cervical cancer remains the most common HPV-related cancer; however, the epidemiology of HPV-related cancers has changed in recent years, leading to an increased role for HPV in head and neck cancers, particularly in high-income countries. At the same time, the introduction of the HPV vaccine has led to a significant reduction in the incidence of genital warts [6,22]. Recent studies have focused on exploring the potential association between HPV and cancers at other anatomical sites, such as breast, lung, gastric, and colorectal cancers. However, the evidence in these areas remains inconsistent, and a definitive causal relationship has not been established [6,23,24]. All cancers associated with HPV infection are shown in Figure 2.

Glandular cervical lesions, such as adenocarcinoma in situ (AIS), although less common than squamous lesions, present significant diagnostic and epidemiological challenges. AIS is strongly associated with HR-HPV, particularly types 16 and 18. According to a recent population-based study [25]. AIS is more commonly diagnosed in non-Hispanic White women, especially at younger ages, and Black and Hispanic women are more often diagnosed at later stages of adenocarcinoma, possibly due to disparities in healthcare access or delayed detection [26]. A separate retrospective case series found that among 47 women diagnosed with AIS, 49% identified as Hispanic, 45% as non-Hispanic White, 4% as Asian, and 2% as Black. Although the sample size was limited, the relatively high proportion of Hispanic patients raised questions about potential differences in HPV exposure, genetic susceptibility, or access to early detection [25] According to studies, women diagnosed with AIS had a higher median age (35 years) compared to those with CIN3 (31 years). AIS cases were more likely to involve non-Hispanic White women, individuals with private insurance, and those ineligible for HPV vaccination. Moreover, women with AIS alone were older than those with AIS combined with CIN (37 vs. 32 years) and were more often ineligible for vaccination (72.3% vs. 51.5%) [27]. An analysis of the National Cancer Database from 2004 to 2017 revealed that Black women with cervical adenocarcinoma were 16% less likely to be diagnosed at an early stage compared with White women. Additionally, women with public insurance were less likely to be diagnosed at an early stage for both adenocarcinoma and squamous cell carcinoma [28]. A population study covering the years 2000–2018 reported that Black women had the lowest incidence of cervical adenocarcinoma but experienced the highest mortality rates and the lowest five-year survival, especially in advanced stages. In contrast, Hispanic women had higher incidence rates but better survival outcomes, possibly due to earlier detection or more effective treatment responses [26]. Some study findings suggest potential genetic differences in tumor biology between ethnic groups. According to a study, Latina women exhibited a higher frequency of missense mutations in ERBB2/ERBB3 genes, while non-Latina White women showed more truncating mutations in KMT2C/KMT2D genes [29]. Moreover, evidence suggests that HPV genotype distribution differs by ethnicity; for instance, HPV35—a high-risk genotype more prevalent among women of African ancestry—has been associated with more aggressive disease progression and may contribute to disparities in cervical cancer burden [30].

During HR-HPV infection, HPV E6 and E7 oncoproteins (viral proteins that promote cell proliferation and inhibit tumor suppressor pathways) integrate into the human genome, impairing DNA repair and cell cycle control [9]. Usually, the host immune system recognizes infected cells by secreting interferons such as IFN-α and IFN-β, which activate the natural immune response including macrophages and dendritic cells. Subsequently, cytotoxic T lymphocytes, activated by interferon gamma (IFN-γ) signals, attack the virus-infected cells, inducing apoptosis [9,19]. In cases of vaginal dysbiosis, this normal immune response is disrupted. Imbalance in the vaginal microbiota, a decrease in the Lactobacillus population, and the resulting increase in pH create a favorable environment for pathogenic bacteria [9,31]. Pathogenic bacteria compromise the protective barrier by producing enzymes such as sialidases that degrade protective mucus and weaken epithelial integrity, promoting the integration of HR-HPV into the host genome. The disruption of these barriers leads to a weakened immune system, resulting in excessive release of inflammatory cytokines (e.g., IL-1β, IL-6, IL-12, TNF-α), chronic inflammation, oxidative stress, and immune exhaustion, all of which interfere with viral clearance and contribute to premalignant changes [9,19,31] and cell cycle control [9]. 

Vaginal *Lactobacillus* has the potential to inhibit the expression of viral oncogenes (E6/E7), reduce cancer cell proliferation, and induce apoptosis. A *Lactobacillus*-dominant vaginal microbiota enhances protective function, improves local immune response, and reduces the risk of progression of HR-HPV infection [9,31,32]. *Lactobacillus* prevents colonization by other bacteria and pathogens by producing lactic acid, hydrogen peroxide, and bacteriocins, which help maintain the integrity of the mucosal barrier and protect against viruses and opportunistic bacteria. Some *Lactobacillus* species secrete biosurfactants that inhibit biofilm formation, bacterial adhesion, and viral entry [19,31,32].

International guidelines advise surgical treatment of high-grade intraepithelial lesions (CIN 2/3) and a surveillance strategy for low-grade lesions (CIN 1). CIN 1 regresses in 80–90% of cases, while CIN 2/3 regress less often and can progress to cancer [16]. However, some CIN 2 lesions also regress, and it is necessary to identify specific prognostic factors that predict lesion regression. One such factor may be the vaginal microbiota [33,34]. The identified risk factors for HPV persistence and progression to dysplasia include HPV genotype (especially HPV 16 and HPV 18), viral load per unit cell, integration of viral DNA into cellular DNA, co-infection with human immunodeficiency virus (HIV), immunosuppression, tobacco use, and factors related to the vaginal microbiome [35].

In 2012, the World Health Organization (WHO) initiated the harmonization of cytological (Pap test, based on the Bethesda System) and histological classifications (biopsy-based), aiming to standardize terminology globally [36]. This effort resulted in an updated definition of cervical intraepithelial neoplasia (CIN) that aligned terminology between histological and cytological findings: LSIL corresponds to CIN 1, HSIL includes CIN 2 and CIN 3, and carcinoma in situ refers to full-thickness epithelial atypia with malignant features [36,37].

Precancerous changes in the cervix are most often caused by long-term infection with oncogenic HPV types, especially 16 and 18 [17]. To help prevent cervical cancer, women undergo screening for early cellular abnormalities using cervical cytology (Pap testing), HPV DNA testing, or a combination of both. Following established screening protocols has been shown to lower cervical cancer incidence by 50–80% and substantially reduce related deaths [37]. Although cytology-based Pap testing has played a key role in reducing cervical cancer incidence, it carries a risk of overdiagnosis and overtreatment, especially for LSIL that may regress. Conversely, HPV DNA testing is significantly more sensitive, particularly for detecting HSIL, but it may identify transient infections in younger women that are unlikely to lead to malignancy [38]. Co-testing (combined HPV and Pap testing) is recommended for women over 30 years of age. This approach provides 60–70% greater protection against invasive cervical cancer compared with cytology alone. Furthermore, recent evidence supports primary HPV screening as a preferred strategy in many countries due to its superior predictive value for HSIL [38,39]. According to current guidelines, cervical cancer screening is recommended for women aged 21 to 65 years [37].

Not all precancerous cervical lesions progress to cancer; however, HSIL carries a substantial risk of progression to invasive cancer if left untreated [11,36]. According to international guidelines, surgical treatment is recommended for HSIL, while a conservative, watchful waiting approach is advised for LSIL, as these frequently regress spontaneously. Therefore, identifying specific prognostic factors that can distinguish between regressive and progressive lesions is crucial. One emerging candidate is the composition of the vaginal microbiota [2].

### 3.2. Factors Influencing the Composition of the Vaginal Microbiota

The vaginal microbiota is formed by vertical transmission during vaginal delivery. From birth to adulthood, the microbiota changes and depends on ethnicity, mode of delivery, breastfeeding, household (e.g., siblings, pets, etc.), antibiotic use, chronic diseases, etc. [35,40]. The vaginal microbiota of reproductive and older women is influenced by the cyclic secretion of estrogen and progesterone throughout the menstrual cycle, menstruation or menopause, use of synthetic hormones for contraceptive purposes, sexual intercourse, hygiene habits, and infections [32,35]. Once the microbiota becomes stable, it can be changed by diet, physical activity level, habits (tobacco, alcohol consumption), and antibiotics use [40]. *Lactobacillus* bacteria have been found to dominate the vaginal microbiota of Caucasian women. In contrast, the microbiota of Hispanic, African, and African American women is less stable and has a greater diversity, and the *Lactobacillus* population is lower [18,32,41]. According to studies, factors such as vaginal douching, antibiotic use, lubricant use, obesity, and smoking adversely affect the vaginal microbiota [32]. Factors such as new sexual partners can also disrupt the microbiota balance. Therefore, it is essential to use protective measures (e.g., condoms), the use of which is associated with a more stable microbiota [42]. Levonorgestrel intrauterine systems (LNG IUSs) and copper intrauterine devices (CuIUDs) reduce the amount of *Lactobacillus* and increase the risk of BV [32,42]. Stress is also a very important negative factor; a five-point increase in stress on the PSS-10 stress scale was associated with a 26% higher risk of BV [42]. Meanwhile, estrogen is considered a beneficial factor because it promotes the accumulation of glycogen in epithelial cells, which is a food source for *Lactobacillus*, thereby maintaining an acidic environment. Estrogen replacement therapy in postmenopausal women also helps restore *Lactobacillus* levels [35]. Combined oral contraceptive pills (OCPs) support the growth of hydrogen peroxide-producing *Lactobacillus* and lower the risk of bacterial vaginosis recurrence [32]. It is not clear whether genetic factors influence the vaginal microbiota and precancerous cervical lesions, as data in this area are limited. A twin study suggested that genetic factors may influence the vaginal microbiota: the similarity of the microbiota was highest between monozygotic twin pairs, followed by sister and mother–daughter pairs, and lowest among unrelated subjects. It has been observed that the composition of the vaginal microbiota can be inherited from the mother, like the intestinal microbiota. Still, heredity only influences the extent to which a newborn girl is infected with bacteria from her mother at birth. However, environmental factors, including diet and lifestyle, are more significant in forming microbiota composition, even among genetically identical individuals [35].

The gut–vaginal microbiota axis also affects the vaginal microbiota, with approximately 30% of bacterial species being shared between them. A large diversity of bacteria in the gut is thought to be a sign of good health. At the same time, a healthy vaginal microbiota is characterized by a predominance of one or two different *Lactobacillus* species [19]. *Lactobacillus* dominance in both the rectum and vagina is associated with a reduced risk of BV [43,44]. Gut bacteria, such as *Ruminococcus* and *Clostridium*, break down complex carbohydrates and produce short-chain fatty acids (SCFAs) such as butyrate. They promote the differentiation of regulatory T cells, maintain anti-inflammatory responses, and suppress inflammation not only in the gut but also in the vagina [45]. The gut microbiota participates in estrogen metabolism through the so-called enterohepatic circulation. Estrogens regulate glycogen production in the vaginal epithelium. Glycogen is the main source of nutrients for *Lactobacillus* bacteria [43]. If estrogen metabolism is disrupted due to an imbalance in the intestinal microbiota, this can negatively affect the dominance of *Lactobacillus* and the stability of the vaginal microflora [43,44].

### 3.3. Composition and Functions of the Vaginal Microbiota

Until recently, little was known about the microbiome, but in the past decade, due to improved technologies in microbiology, research on the microbiome has increased significantly. The vaginal microbiota is defined as the species and communities of bacteria (commensal, symbiotic, pathogenic) or microorganisms, and the microbiome includes the genomes or genetic information of these microbes, including bacteria, fungi, viruses, and microbes, defining the functional potential of these microorganisms [1,35]. The normal vaginal microecosystem is mainly composed of *Lactobacillus* species, although various bacteria, such as *Streptococci*, and other microorganisms, such as mycoplasma and viruses, can also be found, which can be part of the normal microbiota and not cause infections [46,47]. Healthy vaginal microbiota of reproductive-age women are dominated by one or two of four *Lactobacillus* species: *L. crispatus*, *L. gasseri*, *L. jensenii* and *L. iners*. *L. crispatus*, *L. gasseri*, and *L. jensenii* are now believed to maintain a homeostatic non-inflammatory environment. *L. iners* is a transient species that usually colonizes when the balance of vaginal environment is disrupted and is generally not that helpful against vaginal dysbiosis and BV [35,48]. Homeostatic *Lactobacillus* perform various main protective functions; they break down glycogen to lactic acid, reducing vaginal pH and creating an acidic environment, they inhibit pathogen colonization by producing bacteriocins and biosurfactants, they compete with other microorganisms for nutrients and sites of attachment to the vaginal epithelium, and they modulate the immune environment of the vagina to protect their host [1,35]. Vaginal microbiota are generally classified among five community types (CSTs) based on the predominant *Lactobacillus* species. Based on this classification, the authors reported that CST I, II, and V communities were significantly associated with healthy vaginal microbiota dominated mostly by *L. crispatus*, *L. gasseri*, and *L. jensenii*. In contrast, CST IV, dominated by anaerobic bacteria, was associated with decreased *Lactobacillus*, BV, and CIN [20,41,49]. CST I is characterized by a predominance of *Lactobacillus crispatus*, low microbial diversity, and a notable presence of *Lactobacillus acidophilus*. CST II includes predominantly *L. gasseri* and *L. johnsonii*. CST III is dominated by *L. iners*, with *L. acidophilus* also present. CST V is dominated by *L. jensenii* with high levels of *L. acidophilus* and *L. iners*, while maintaining low microbial diversity. CST IV is defined by a diverse microbial community, with an abundance of *G. vaginalis* and *M. genomosp type 1* (*Megasphaera genomosp. type 1*), and a decrease in *Lactobacillus* species, except for *L. iners* [49]. During cervical carcinogenesis caused by HR-HPV infection, the microbiota mainly exerts pathogenic or protective effects, influencing the development of lower genital tract inflammation, immune response, oxidative stress, cellular DNA, and tumor metabolism [9]. CST types I, II, V perform a protective function within the vaginal microbiota, CST III performs both a protective and pathogenic function, while CST IV has a pathogenic function. It has been established that with increasing diversity of the vaginal microbiota and the predominance of CST type IV, cervical lesions progress [47]. It is essential to highlight that longitudinal studies of the vaginal microbiome show that the structure of the bacterial community is dynamic and changes depending on the factors listed above [2].

However, with the growth of metagenomic studies and the integration of more advanced analyses (e.g., metatranscriptomics, metabolomics), it has been proposed to refine the classification by taking into account functional changes, not just taxonomic composition, subtypes within certain CSTs (especially CST IV), and transition states between stable forms of CSTs [50]. More recent studies have proposed additional CST variants, particularly related to *Gardnerella vaginalis* subtypes. This inconsistency complicates comparisons across studies. To enhance classification, the vaginal community state type nearest centroid classifier (VALENCIA) tool was developed to enable standardized CST assignment. Most samples still clustered into the five classic CSTs, with subtypes identified primarily within CST I, III, and IV [49]. CST I-A is characterized by the dominance of *Lactobacillus crispatus*, low levels of *L. acidophilus*, and low microbial diversity. In CST I-B, *L. crispatus* also predominates, but with a significantly higher abundance of *L. acidophilus* compared with CST I-A. CST II is dominated by *L. gasseri* and *L. johnsonii*, with increased diversity of other bacterial taxa [51]. CST III-A shows a predominance of *L. iners* and low microbial diversity (being predominant in patients with vulvovaginal candidiasis, *Chlamydia trachomatis*, and herpes simplex virus infections), whereas CST III-B is also *L. iners*-dominant but with a notable presence of *L. acidophilus*, more prevalent in patients with BV. CST IV-A is characterized by a diverse microbial community marked by a high abundance of *Gardnerella vaginalis* and low levels of *Megasphaera genomosp. type 1*, and it is most commonly associated with BV [49,51]. In contrast, CST IV-B is primarily dominated by *Megasphaera genomosp. type 1*, also contains abundant *G. vaginalis*, and shows a marked reduction in *Lactobacillus* species, except for *L. iners*. This subtype is strongly linked to inflammatory conditions, persistent HPV infection, and CIN 2 [52]. CST IV-C represents a transitional microbiota state typically dominated by *Lactobacillus iners*, with increasing anaerobic overgrowth, and includes subtypes IV-C1, IV-C2, IV-C3, and IV-C4, characterized by the predominance of *Prevotella*, *Enterococcus*, *Bifidobacterium*, and *Staphylococcus* species, respectively [51]. Finally, CST V is dominated by *L. jensenii*, with high levels of *L. acidophilus* and *L. iners*, while maintaining low microbial diversity [12].

Recent research has revealed associations between vaginal CSTs and ethnicity. Roachford et al. found that CST I (*Lactobacillus crispatus*) was most prevalent in African American women (50%) but less common in African Kenyan (9.7%), Afro-Caribbean (11%), Asian Indonesian (19%), and Caucasian German (18%) women. CST II was rare, found only in 2.8% of Asian Indonesian women. CST III was distributed among all groups, ranging from 46% to 14%. CST IV subtypes were more frequent in non–African American populations. CST V was not observed in any group. These patterns underscore the relevance of ethnic variation in CST distribution and its potential impact on HPV-related cervical disease risk [53]. Vancuren et al. reported that Black and Latino women had a significantly higher prevalence of CST IV compared with White and Asian women. CST III was also more common among Black, Asian, and Latino women [54]. In addition to differences in CST distribution among ethnic groups, variations in vaginal pH have also been observed, with Hispanic and Black women having higher vaginal pH levels compared to Asian and White women [55].

### 3.4. The Role of Lactobacillus Iners

Not all *Lactobacillus* species protect the vaginal microbiota equally. Studies have shown that *L. iners* has lower resistance to infection and may promote dysbiosis and HPV persistence [56]. *L. iners* produces very little H_2_O_2_, which has antibacterial properties and helps to inhibit the growth of *Atopobium vaginae*, *Prevotella* spp., and *Gardnerella vaginalis*. In the presence of low H_2_O_2_, these bacteria can multiply more quickly, causing vaginal dysbiosis and BV [32]. *Lactobacillus* species can synthesize different isomers: D-isomer and L-isomer of lactic acid. Studies show that *L. crispatus* mainly produces D-isomer lactic acid, which has a stronger bacteriostatic effect and is more effective in maintaining an acidic vaginal pH. *L. iners* produces only the L-isomer lactic acid, which is less inhibitory to pathogens than D-isomer lactic acid and therefore is unable to maintain a low pH, which may contribute to the development of BV [35,48]. A study by Xu et al. revealed that the abundance of *L. iners* was significantly correlated with HPV infection and CIN. The shift from CST I to CST III was most pronounced in HPV-positive women, indicating that *L. iners* may be the dominant species during HPV infection [56]. During menstruation, when the vaginal pH becomes less acidic due to exposure to blood, *L. iners* remained dominant. However, the abundance of other *Lactobacillus*, such as *L. crispatus*, was significantly reduced [32]. The ability of *L. iners* to attach to the vaginal epithelium via fibronectin-binding adhesion proteins helps it survive despite exposure to BV or antibiotics. *L. iners* is more commonly found after BV treatment than during active BV, suggesting that it may be an intermediate bacterium between a healthy microbiota and a dysbiosis state [56]. The *L. iners* genome contains genes for a pore-forming toxin, inerolysin, which has structural and functional similarities to vaginolysin produced by *Gardnerella vaginalis*. Thus, *L. iners* is adaptable to changing conditions and exhibits features of both beneficial *Lactobacillus* and vaginal pathogens [9].

### 3.5. Altered Vaginal Microbiota and Pathogenic Mechanisms of BV

The main function of healthy vaginal microbiota *Lactobacillus* spp. is to prevent colonization by BV-associated bacterial species by maintaining acidic pH and bacteriocin production [32]. This is important in maintaining the barrier function of the cervical epithelium, which prevents HPV infection from entering basal keratinocytes. Dysbiosis of the vaginal microbiota involves disturbance of the balance of normal microorganisms [57]. When BV-associated anaerobes colonize, they produce enzymes and metabolites that can disrupt the cervical epithelium barrier and enhance the entry of HPV. The bacteria that cause BV alter the vaginal environment, weaken defense mechanisms, and promote inflammation, thereby creating favorable conditions for HPV infection to persist and progress to CIN or even cancer [32,57]. BV occurs in the reproductive age group, with 15–50% of women having a vaginal infection. Women manifest with vaginal discharge (with exfoliated epithelial cells and Gram-variable polymorphic bacteria attached to their surfaces), a characteristic odor, itching, or an increased vaginal pH ≥ 4.5 [56]. There is no standard definition of BV, but it is widely accepted that it is a process associated with alterations in the composition of the vaginal microbiota, such as a decrease in *Lactobacillus* and colonization by anaerobic microorganisms, mainly *Gardnerella vaginalis*, *Prevotella* spp., *Atopobium vaginae*, *Bacteroides*, *Peptostreptococcus*, *Smobilattrichus hominisa*, *Mycoplasmattrichus* spp., *Mobiluncus*, *Sneathia*, *Leptotrichia*, *Ureaplasma*, etc. [9,32,35,56]. Statistical analysis revealed a significant association between bacterial vaginosis (BV) and HPV persistence. Studies have reported odds ratios between 1.8 and 3.4 (*p* < 0.05), indicating increased risk of persistent HR-HPV infection among BV-positive women [2,3]. Studies have shown that BV is associated with pathogenic mechanisms such as an increase in vaginal pH, the formation of a polymicrobial biofilm, damage to the epithelial barrier, promotion of inflammation, and suppression of the immune system. During BV, the dominant anaerobic bacteria reduce lactic acid production and inhibit the activity of *Lactobacillus* species, so pH increases to >4.5, which creates favorable conditions for the survival of HPV [2,56]. *Gardnerella* spp. have been shown to damage the mucosal barrier, reduce the viscosity of cervical secretions, alter vaginal epithelial homeostasis, and thus promote infections [57]. *Prevotella* and *Bacteroides* spp. produce sialidases, enzymes that degrade mucus and damage the cervical epithelium. These bacteria also produce biological amines that cause oxidative stress, a key mechanism that promotes carcinogenesis and increases the risk of HPV infection [11]. *Gardnerella* spp. and other anaerobic bacteria of BV participate in synergistic interactions and can form a polymicrobial biofilm, which is considered one of the factors determining the chronicity and recurrence of the disease. The biofilm has a complex internal architecture that helps to tolerate adverse conditions and protects against antimicrobial molecules (e.g., antibiotics, antiseptics, etc.) and the human immune response. Due to the formation of a polymicrobial biofilm upon contact with sexually transmitted infections (STIs) pathogens *C. trachomatis*, *M. genitalium*, *N. gonorrhoeae*, *T. vaginalis*, HIV, or HPV, patients with BV are twice as likely to contract STIs compared with women without BV [57]. Anaerobic bacteria of BV reduce the innate immune response, suppress the apoptosis process, which slows wound healing, and create favorable conditions for HPV infection to reach basal cells and integrate. For this reason, women with BV have reduced levels of an important innate immune protein, secretory leukocyte protease inhibitor (SLPI), which protects the mucosa from infection [11]. This is considered a potential mechanism for cervical carcinogenesis [9]. BV-associated bacteria correlate with pro-inflammatory cytokines (IL-6, IL-8, TNF-α), which increase oxidative stress and promote cellular changes, increasing the risk of HPV progression to higher grade CIN [11]. Vaginal pro-inflammatory cytokines are higher in women with dysbiosis and can lead to chronic inflammation, which is a well-known factor in carcinogenesis of many tissues in the body [32]. Dysbiosis creates an inflammatory environment, activating macrophages, neutrophils, and epithelial cells, which produce large amounts of hydroxyl radicals (OH^−^) and reactive oxygen species (superoxide anion (O_2_^−^), etc.). These molecules tend to cause oxidative stress, which then damages DNA (causing double-strand breaks), proteins (oxidizing and inactivating enzymes), and lipid membranes (promoting cell damage). In the presence of DNA double-strand breaks and oxidative stress, HPV E6 and E7 oncoproteins block pRb, degrade the p53 protein, and activate HPV integration into the host genome [2,9,32]. *Prevotella* is thought to promote cell proliferation, contribute to the development of cervical lesions, and potentially disturb immune regulation by influencing several key molecular pathways in cervical cells. These include the *C-myc* oncogene, which drives uncontrolled cell growth; Toll-like receptor 4 (TLR4), involved in immune recognition of microbial components; nuclear factor-kappa B (NF-κB), a transcription factor that regulates inflammation and immune responses; and human telomerase reverse transcriptase (hTERT), an enzyme that maintains telomere length and is often upregulated in cancer cells [9]. It has been found that the vaginal microbiota of HPV-infected women and postmenopausal women is much more diverse, characterized by an abundance of anaerobic bacteria and a decrease in the amount of *Lactobacillus* [58]. Although the studies described suggest that the vaginal microbiota may have a significant impact on the clearance, persistence, and progression of HPV infection to CIN, it is still unclear whether HPV infection promotes dysbiosis of the vaginal microbiota or vice versa, i.e., dysbiosis helps HPV infection to persist [10,56]. It is thought that anaerobic bacterial genera such as *Atopobium*, *Sneathia*, and *Gardnerella* may be markers of HPV infection and progression of cervical precancerous lesions. However, their mechanisms of action are not fully understood. Recent in vitro studies have suggested that *Peptoniphilus lacrimalis* and *Fusobacterium nucleatum* can contribute to inflammation and changes in the cervical epithelium. This would support the idea that changes in the microbiota may be an important factor in the progression of CIN [47]. HPV E6/E7 oncogene expression becomes more active when the quantity of *Lactobacillus* decreases and the abundance of genera such as *Sneathia* and *Megasphaera* increases in CIN or *Peptostreptococcus* and *Enterococcus* increase in cervical cancer [20]. HPV-infected women have higher levels of *L. iners* than healthy women, and the dominance of *L. crispatus* in the vagina increases the viscosity of cervical mucus and promotes viral shedding, helping to prevent persistent infection [56]. *Atopobium* spp. dominance in the vaginal microbiota is associated with HPV persistence and *L. gasseri* is associated with HPV clearance [59]. It has been found that the levels of vaginal anaerobic bacteria *Peptostreptococcus anaerobius*, *Gardnerella vaginalis* and *Porphyromonas uenonis* are increased in women with CIN and cervical cancer [60]. *Acinetobacter*, *Prevotella*, and *Pseudomonas* have been found to correlate with persistent HPV infection, suggesting that women with high levels of *Prevotella* and *Pseudomonas* have more difficult HPV clearance [60,61]. Bacteria such as *Sphingomonas* and *Phyllobacterium* have also been associated with persistent HPV infection, but further studies are needed to confirm this association [61]. During HPV progression, there is a strong link between potential biomarkers such as *Sneathia* and *Delftia*, found in CST IV and CST II community types, and chronic inflammation with excessive cytokine expression. A more detailed analysis of these factors could be useful for developing new treatment strategies and preventing disease progression [3]. Not only bacteria contribute to HPV ubiquitination and cellular changes; *Sporidiobolaceae* and *Saccharomyces* have been identified as potential fungal markers in atypical squamous cells of undetermined significance (ASCUSs). An important fungal marker associated with HR-HPV infection is also *Malassezia*. *Anelloviruses* are found in significantly larger quantities in LSIL and cervical cancer. Increased abundance of anelloviruses correlates with decreased *Lactobacillus* and CST IV. Based on these data, we can conclude that anelloviruses may be markers of carcinogenesis and cervical dysplasia [9]. Studies have shown that the predominance of *L. iners* promotes the development of BV, while the dominance of *L. crispatus* prevents the development of BV [62]. Based on the reported studies, it can be concluded that BV may be associated with a higher prevalence and persistence of HPV infection and the development of CIN. Recent studies using next-generation sequencing methods indicate that increased diversity of the vaginal microbiota, together with a reduced relative *Lactobacillus* spp., is involved in the persistence of HPV and the progression of CIN and cancer [2,63]. In conclusion, the restoration of microbiota homeostasis could be a way to eliminate HPV infection, since an intact cervical epithelium, maturity, and a small transformation zone area create an unfavorable environment for infection and viral persistence [9,64].

### 3.6. The Role of Vaginal Microbiota in Pregnancy Outcomes and Preterm Birth

Evidence from multiple studies suggests that high-risk HPV infection in conjunction with pregnancy may exert a synergistic effect on the vaginal microbiota, promoting increased microbial diversity and compositional complexity [65,66]. During pregnancy, the protective role of the vaginal microbiota against infections is enhanced, largely due to hormonal changes that promote increased dominance of *Lactobacillus* species [67] Persistent HPV infection may further disturb the vaginal environment by reducing *Lactobacillus* abundance, increasing vaginal pH, and promoting chronic inflammation. These changes weaken epithelial defenses and create conditions that may elevate the risk of preterm labor and other complications [66]. Dysbiosis and BV have been shown to play a critical role in various pregnancy outcomes, including spontaneous preterm labor, spontaneous abortion, amniotic infection, preeclampsia, in vitro fertilization, embryo transfer, and premature rupture of membranes (PROM) [68,69]. PROM is associated with vaginal dysbiosis, which raises the risk of neonatal and maternal complications such as reduced birth weight, early-onset neonatal sepsis, shorter gestational age, urinary tract infections, endometritis, and chorioamnionitis [68]. It has been found that pathogens such as *Megasphaera*, *Prevotella*, *Mycoplasma hominis*, and *Ureaplasma urealyticum* are associated with lower gestational age, risk of preterm birth, and birth weight, while species like *Streptococcus*, *Peptoniphilus*, and *Dialister* are linked to membrane rupture [68,70]. A vaginal CST characterized by low *Lactobacillus* levels and increased microbial diversity—including higher amounts of *Bacteroides*, *Halomonas*, *Bacillus*, *Staphylococcus*, *Escherichia*, and *Acetobacter*—has been linked to a greater risk of missed miscarriage [71]. Premature rupture of membranes (PROM), ascending infections, and microbial invasion of the amniotic cavity are recognized risk factors for the onset of preterm birth [69]. According to Gudnadottir et al., women with a low levels of beneficial bacteria (*Lactobacillus*) in the vaginal microbiome had significantly higher risk of preterm birth compared with those dominated by *Lactobacillus crispatus* (OR 1.69; 95% CI 1.15–2.49) [69]. On the other hand, an increased abundance of *Lactobacillus* species is thought to offer protective benefits against preterm birth. These findings highlight the potential value of targeting the vaginal microbiome as a preventive approach to reduce the risk of spontaneous preterm birth [70]. Based on the meta-analysis by Nijibizi et al., HPV infection was found to be significantly linked to several adverse pregnancy outcomes. These include an increased risk of preterm birth (aOR 1.50; 95% CI: 1.19–1.88), early rupture of membranes before labor (aOR 1.96; 95% CI: 1.11–3.45), general membrane rupture (aOR 1.42; 95% CI: 1.08–1.86), restricted fetal growth (aOR 1.17; 95% CI: 1.01–1.37), low birth weight (aOR 1.91; 95% CI: 1.33–2.76), and a higher risk of fetal death (aOR 2.23; 95% CI: 1.14–4.37) [72].

While only 2% of European pregnant women were reported to have CST IV dominated by *Gardnerella vaginalis*, nearly 50% of African women in a similar study exhibited this microbial profile [73].

### 3.7. Relationship Between Vaginal Microbiota, HPV Infection, and Cervical Dysplasia

Studies have shown that women with a *Lactobacillus*-dominated microbiota are associated with a lower risk of developing cervical precancerous lesions [49]. However, the available data are insufficient to determine the precise role of *Lactobacillus* species in the development of cervical precancerous lesions [41]. 16S rRNA sequencing is a targeted method that uses next-generation sequencing technology to analyze bacterial and archaeal phylogenetic markers (16S rRNA genes), allowing rapid and efficient sequencing of DNA or RNA sequences on a large scale [19,74]. Shotgun metagenomic sequencing provides higher taxonomic resolution, allowing the study of bacteria, fungi, protozoa, and viruses, and the analysis of functional properties of the microbiota [47,75]. Taxonomic programs applying this method can be used to investigate phylogenetic relationships between detected sequences and known microorganisms. Functional metagenomics methods focus on identifying functional genes and novel proteins that contribute to the activity of microbial populations [74]. Quite a few studies have been written analyzing the composition of the microbiota and its strains in relation to HPV and precancerous lesions, but the research methods used differ. Although the results of most of these studies as described by their authors coincide, it is worth noting that shotgun metagenomic sequencing has a higher taxonomic resolution compared with 16S rRNA. Genetic changes detected by the shotgun approach may become early biomarkers that more accurately predict the course of infection [76]. A summary of cervical microbiota composition studies conducted using shotgun metagenomic sequencing and 16S rRNA methods in women with HPV, LSIL/HSIL, and cervical cancer is presented in Table A1.

The information of most researchers shows that in normal cervical epithelium and the absence of HPV infection, *Lactobacillus* spp., especially *L. crispatus*, dominate [1,47,77]. Norenhag et al. revealed that the microbiota of women with dysplasia had more functional pathways with negative effects than the microbiota of healthy women. *G. vaginalis* contributes to the promotion of peptidoglycan biosynthesis and L-alanine biosynthesis pathways, which are associated with cervical cancer. In a group of healthy women, *L. crispatus* participated in the regulation of L-lysine, flavin, L-threonine, and L-lysine/L-threonine/L-methionine biosynthesis. L-lysine/L-threonine/L-methionine biosynthesis is associated with cancer preventive functions [47]. Zhou et al. found familiar changes in the vaginal microbiota in women with BV, CIN and cervical cancer. Increased abundance of *Clostridium*, *Sneathia*, *Megasphaera*, and *Prevotella* genera and decreased abundance of *Lactobacillus* were found in these diseases [78] A study by Kyrgiou et al. found that for women whose vaginal microbiota were not dominated by *Lactobacillus*, the regression of cervical precancerous lesions was slower. In that study, the microbiota of women whose CIN had not regressed by 12 months were enriched with *Prevotella*, *Megasphaera*, and other BV-associated bacterial species [11]. A study by Liu et al. found that the abundance of *Lactobacillus* decreased with disease progression, while the abundance of *Gardnerella* and *Prevotella* increased. It was found that CST IV dominance correlated with CIN [20]. Zheng et al. found that vaginal microbiota dysbiosis increased unmethylated cytosine–phosphate–guanine (CpG) clusters—specific DNA regions that can activate immune responses when unmethylated—leading to increased expression of Toll-like receptor 9 (TLR9), a receptor involved in recognizing microbial DNA and initiating innate immune responses. TLR9 protein levels and microbiota diversity increased with cervical lesion progression. The expression of this protein was higher in HPV 16-positive women than in HPV 16-negative women in the normal cervix, CIN 1, and CIN 2/3 groups [79]. Hu et al. found that HPV-infected patients exhibited increased nucleotide biosynthesis and DNA replication, aiding viral replication. Patients with CIN and cervical cancer had higher levels of signaling proteins associated with proliferation and inflammation. At the same time, healthy controls showed improved sugar metabolism, including pyruvate, starch, and sucrose metabolism, indicating that their microbiome was more homeostatic and not inflammatory [76]. Kwon et al. found that cervical cancer patients had increased cell cycle control and carbohydrate metabolism but decreased defense and transcription. Analysis revealed increased peptidoglycan biosynthesis in cancer patients, and increased methane, lysine metabolism, and kidney cancer pathways were more active in the CIN 2/3 group. The microbiota of healthy control patients was characterized by increased activity of toxin degradation. and biofilm formation pathways [80]. In the study conducted by Fang et al., four significant genes were identified: N-acetylglucosamine-6-phosphate deacetylase, sugar-specific component IIA, small subunit ribosomal protein S21, and large subunit ribosomal protein L33, which were characterized by their abundance in HPV-uninfected control women. It was also found that the abundance of the phosphotransferase system, a major mechanism for bacterial carbohydrate uptake, was positively associated with *L. crispatus* and *L. iners* but negatively correlated with *G. vaginalis* [81]. Yang et al. reported that metabolic processes such as carbohydrate and amino acid metabolism, membrane transport, and signal transduction were more active in the vaginal microbiota of women with HPV 16 infection. In the control group, glycan biosynthesis and metabolism were more pronounced. Stronger glycan biosynthesis in the normal vaginal microbiome may protect against microbiota imbalance and HPV infection Three species were highly abundant in the HPV16-positive group, including *Atopobium vaginae* (*p* = 2.66 × 10^−8^, Wilcoxon rank sum test), *Peptostreptococcus anaerobius* (*p* = 2.79 × 10^−8^, Wilcoxon rank sum test), and *Candida (p* = 2.54 × 10^−6^, Wilcoxon rank sum test) [75]. Kwon et al. observed atypical bacteria such as Pseudoalteromonas and Psychrobacter in a group of healthy women. According to a study by Fan et al. an increase in vaginal microbial diversity, a decrease in the relative abundance of cyanobacteria and *Lactobacillus*, and an increase in the relative abundance of dialyser, peptonephila, and other various bacteria were found in cervical cancer patients. The biomarker for the normal group was *Varibaculum*, that for the HR+HPV group was *Saccharopolyspora*, and the biomarkers for the cervical cancer group were *Proteobacteria*, *Corynebacterium*, *Coprococcus*, *Peptococcus*, and *Ruminococcus*. Several authors, such as Molina et al. and Ma et al., have also reported other bacteria, not necessarily classified as healthy vaginal microbiota, such as *Lactobacillus acidophilus*, *Gardnerella*, *Prevotella*, *Fannyhessea vaginae*, which can be found even in the absence of HPV [49,82]. In low-grade intraepithelial neoplasia (LSIL/CIN1), the main bacterium identified by all authors is *Gardnerella vaginalis*. The authors also noted that in addition to *Gardnerella vaginalis*, *Prevotella bivia*, *L. iners*, *Peptoniphylus lacrimalis*, and *Megasphaera* sp. are found in this condition [60,83]. Additionally, some authors, such as Wu et al. and Ma et al., found increased bacteria of the genus *Snethia* [82,84]. This is compatible with previous data indicating that this bacterium may be associated with early cervical changes. Less frequently mentioned bacteria have also been found and could become potential biomarkers, such as *Saccharopolyspora* [46]. Liu et al. found bacteria such as *Ercella*, *Bacillus*, *B. Lautia*, and *Terrisporobacter.* It has been shown that the diversity of the microbiota increases in CIN 2/3 and HSIL [85]. So et al. and Teka et al. reported a particularly high abundance of *L. iners* [60,83]. Molina et al., Ma et al., and Liu et al. also observed a high prevalence of *Megasphaera* in CIN 2/3 lesions, suggesting that this bacterium may be particularly characteristic of this lesion type [49,82,85]. Liu et al., Zheng et al., and Norenhag et al. also found that high-grade intraepithelial neoplasia (HSIL/CIN 2-3) was associated with an abundance of *Gardnerella vaginalis*, a greater diversity of the microbiota, and anaerobic bacteria. In addition, less common bacteria such as *Alloscardovia omnicolens*, *S. aureus*, *Staphylococcus*, and *Candidatus Endolissoclinum* were also found [20,47,79]. Thus, in summary, it can be concluded that in normal epithelium, *Lactobacillus* spp. dominates, especially *L. crispatus*, which is indicated by most authors. In the presence of CIN and the progression of precancerous lesions, the abundance of anaerobic bacteria increases. The unique bacteria found, such as *S. aureus*, *Candidatus Endolissoclinum*, indicate that the composition of the microbiota may have individual differences, depending on the population of the subjects and environmental factors.

**Figure 3:**
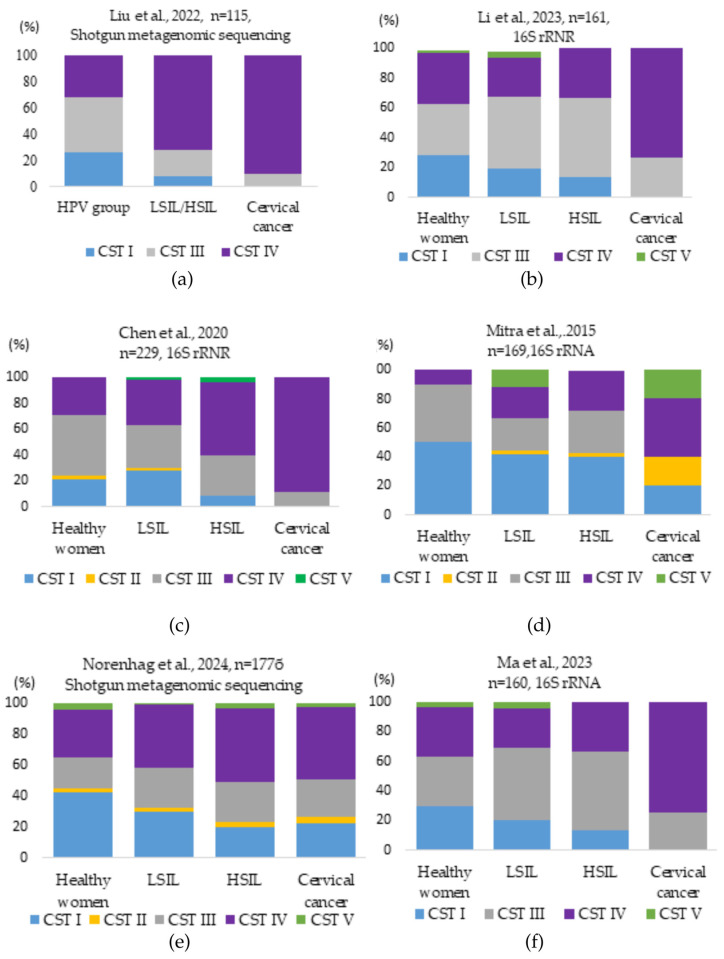
Vaginal microbiota bacterial CST types identified in different control groups in studies conducted by different researchers: (**a**) Liu et al. investigated the dominance of CST I, II, and III positively associated with normal vaginal microbiota. In the HPV group, CST I and CST III accounted for 26.47% and 41.18%. In the LSIL/HSIL and cervical cancer groups, CST I (7.5% and 0%) and CST III (20.00% and 9.76%) significantly decreased, while CST IV (72.50% and 90.24%) increased [20]; (**b**) Li et al., studied that CST I decreased from 28.3% in the healthy group to zero in the cervical cancer group. CST III (26.9%) and IV (73.1%) were prevalent in the cervical cancer group [86]; (**c**) Chen et al., CST analysis of vaginal bacteria revealed that the samples from the healthy patient group were mostly classified as CST type III. CST types IV and III dominated in the HPV-positive groups. CST IV increased in LSIL (35.29%), HSIL (56.52%), and cancer (88.89%) groups [19]; (**d**) Mitra et al. analysis showed that the frequency of CST IV was 2-fold higher in women with LSIL, 3-fold higher in HSIL, and 4-fold higher in women with cervical cancer, while the frequency of CST I decreased by half with increasing disease severity [2]; (**e**) Norenhag et al.’s analysis showed that in the healthy control group, CST I was dominant (42.4%), while CST IV and III were present at 31% and 24.3%. The most common CST among women with dysplasia was CST IV (44.6%), followed by CST III (26.0%), and CST I (24.3%) [47]; (**f**) Ma et al.’s analysis showed that the percentage of CST I decreased from 30% in the healthy patient group to zero in the cervical cancer group. CST III increased from 33.3% in the healthy patient group to 52.8% in the HSIL group. CST III and IV dominated (25.9% vs. 74.1%) in the cervical cancer group [82].

Figure 3 reports the relative abundance (%) of major CST groups in vaginal samples from healthy controls and women from HPV-positive, LSIL/HSIL, and cervical cancer groups, based on studies conducted by different researchers.

As can be seen, CST I consistently decreased with the progression of HPV infection and cervical disease. Significantly, in all studies, the level of CST IV (BV) increased with HPV infection progression and became dominant in cases of cervical cancer [2,13,23,43,49].

Summarizing the studies of the authors discussed above, it can be stated that the normal cervix is dominated by *Lactobacillus* (*L. crispatus*, *L. gasseri*, *L. iners*, *L. jensenii*), which guarantee an acidic environment (pH 3.8–4.5), a mucosal barrier, and active immune cell activity [19,49,87]. In healthy women, *L. crispatus* and *L. gasseri* dominate in over 70% of vaginal microbiota profiles. *L. iners* appear more frequently in transitional or dysbiotic states [31,88]. CST I, typically dominated by *L. crispatus*, is present in 42.4% of healthy women, but drops to 0% in cervical cancer patients, while CST IV, rich in anaerobes like *Gardnerella* and *Sneathia*, increases from 31% in healthy individuals to 90.2% in women with cancer [9,47]. With a predominance of *L. crispatus*, *L. gasseri*, and *L. jensenii*, HPV infection often regresses without causing intraepithelial changes [31]. CIN 1, with favorable microbiota and a good immune response, is also reversible. However, the dominance of *L. iners* is associated with the persistence of the HPV virus [1,35]. During the persistence of HPV infection, *L. crispatus* and *L. gasseri* decrease, but *L. iners* and other anaerobic bacteria increase; as a result, pH increases to >5.0, therefore, the vaginal mucosal barrier and the host immune response are weakened [1,2]. In CIN1, dysbiosis of the microbiota (*L. iners*, *Sneathia* spp., *Megasphaera elsdenii*) is observed, the immune response is suppressed, with decreased activity of Langerhans cells (LCs), dendritic cells (DCs), natural killer (NK) cells, and T lymphocytes, along with an increase in regulatory T cells (Tregs) and M2-type macrophages, which are associated with immunosuppression [31,89]. In CIN 2/3, *Gardnerella vaginalis* and *Sneathia* spp. became more prevalent, with CST IV dominance reported in 73–88% of affected women [49,82]. During this stage, downregulation of immune signaling pathways such as JAK/STAT (Janus kinase/signal transducers and activators of transcription) and NF-κB (nuclear factor kappa-light-chain-enhancer of activated B cells), as well as tumor suppressor gene inhibition, was also observed. In cervical cancer, immune tolerance is established, and the dominance of pathogenic bacteria such as *Fusobacterium* spp. and *Gardnerella* spp. promotes tumor progression [89].

### 3.8. Potential for Modeling Vaginal Microbiota to Reduce the Risk of Cervical Lesions: The Role of Probiotics, Antibiotics and Vaccination

Consequently, combining probiotics and antibiotics is increasingly considered a potential strategy to reduce the risk of CIN [90]. As previously mentioned, BV is one of the primary conditions contributing to vaginal dysbiosis and is strongly associated with persistent HR-HPV infection [32]. In women with BV, the vaginal microbiota is highly diverse, and treatment typically involves antibiotics like metronidazole or clindamycin [90]. Studies show that within 12 months after a 7-day course of oral metronidazole, BV recurs in 58% of cases, and abnormal vaginal flora in 69% of cases [91]. The probiotic LACTIN-V, containing the *Lactobacillus crispatus* CTV-05 strain, significantly reduced BV recurrence following antibiotic (metronidazole or clindamycin) therapy. Thirteen weeks after treatment, BV recurrence was observed in only 39% of women in the LACTIN-V group compared with 54% in the placebo group [92]. In a study by Liu et al., intravaginal administration of *L. crispatus* chen-01 led to a 12.13% higher HPV clearance rate after 6 months compared with the placebo group and significantly improved both inflammatory and cytological outcomes [93]. However, other studies, such as that by Yu-Che Ou et al., found no significant effect on HPV clearance using *Lactobacillus rhamnosus* GR-1 and *Lactobacillus reuteri* RC-14, with only a 3.9% difference in clearance rates between the intervention and control groups [94]. In a randomized controlled trial by Dellino et al., women who received oral *L. crispatus* M247 showed a higher rate of cytological lesion regression (60.5% vs. 41.3%). However, overall HPV clearance did not differ significantly between the groups (15.3% vs. 9.3%) [95]. In summary, there is currently no conclusive clinical evidence that modulation of the vaginal microbiota with probiotics or antibiotics alone can prevent cervical precancerous lesions. However, preliminary findings suggest a potential benefit, especially when used with other preventive strategies [94].

A pivotal study in women aged 16–26 years showed that the nine-valent HPV vaccine (9vHPV) provided statistically significant protection against HSIL caused by the targeted HPV types (16, 18, 31, 33, 45, 52, 58) and HPV-related vulvar, vaginal, anal, and oropharyngeal cancers, as well as HPV types 6 and 11, which cause ∼90% of cases of genital warts and recurrent respiratory papillomatosis [17,96,97]. In a long-term Scandinavian follow-up, no HSIL cases related to these types were observed among 1628 women over 12 years. The vaccine showed over 90% effectiveness throughout the follow-up period, demonstrating durable protection for at least 10 years and suggesting sustained efficacy up to 12 years [96]. As shown in several studies, HPV vaccines are most effective when administered before sexual debut [17]. The 9vHPV vaccine demonstrated sustained immunogenicity and effectiveness after 10 years of three doses of 9vHPV vaccination of boys and girls aged 9 to 15 years [98]. Women vaccinated after becoming sexually active may already have been exposed to HPV, limiting the vaccine’s effectiveness [97]. Therefore, WHO and the United States Advisory Committee on Immunization Practices (US ACIP) recommend starting vaccination in early adolescence between 9 and 14 [98,99,100]. Although the nine-valent vaccine targets the most oncogenic HPV types, it does not cover all existing strains. As a result, cervical lesions caused by non-vaccine HPV types can still occur [101]. Low vaccination coverage and missed doses reduce overall population-level protection and limit herd immunity effects [102]. Delayed vaccination reduces efficacy, especially when individuals are vaccinated after initial HPV exposure or early cervical abnormalities have already developed [99].

Currently, the treatment of precancerous lesions induced by HR-HPV is mainly based on surgical treatment, radiation, and chemotherapy [16]. Although evidence remains limited, preliminary studies suggest that HPV vaccination may influence both local immune responses and the vaginal microbiota. Therapeutic HPV vaccines aim to treat existing infections by targeting viral proteins E6 and E7 to stimulate an immune response. Genome editing tools are being investigated to directly disrupt HPV DNA and prevent cancer progression [16]. Therapeutic vaccination against HPV in women with HSIL was associated with an increase in proinflammatory cytokines, such as IFN-γ and IL-6, without significant changes in microbiota diversity [103]. These findings underscore the need for further investigation into the interplay between HPV vaccines, the vaginal microenvironment, and immune modulation [103].

## 4. Discussion

This review summarizes the findings of studies on the role of microbiota and HPV in the development of dysplasia, indicating promising areas for further investigation [13,14]. Many reviewers emphasize that additional studies are needed to confirm these results and more precisely determine the relationship of the microbiota to the mechanisms of HPV infection integration and the development of dysplasia [19,30,49,62]. The detection of distinct CST types in healthy control and HPV-infected samples, along with their connection to HPV persistence, indicates a potential relationship that needs further exploration [14,104]. Vaginal microbiota dominated by non-*Lactobacillus* species or *Lactobacillus iners* was associated with a three- to five-fold higher likelihood of any common HPV and a two- to three-fold higher likelihood of high-risk HPV and dysplasia/cervical cancer compared with Lactobacillus crispatus [47]. Studies have shown that the dominance of *Lactobacillus crispatus* in the vaginal microbiota is associated with a reduced risk of HPV infection, as these bacteria maintain an acidic environment that inhibits the growth of anaerobic bacteria and the development of inflammatory processes [18,30,61]. The incidence of BV ranges from 15% to 50% among women of reproductive age, and is associated with increased vaginal pH (>4.5), biofilm formation, and immune suppression [56,57]. BV may be associated with a 1.8–3.4 times higher risk of persistent HR-HPV infection and CIN development [11]. Studies also revealed that women with cervical cancer were most likely to have a CST microbiota status IV, which is characterized by very high levels of anaerobes [47,49,85]. These results are consistent with previous studies that highlighted that *Lactobacillus* spp. are essential microorganisms that ensure the stability of the vaginal microbiota [46,47,75]. Furthermore, it is important to note that the composition of the microbiota can vary depending on various factors, such as ethnicity, genetics, smoking, HIV infection, sexual behavior, and sexually transmitted infections [15,58,64].

This literature review includes recent studies examining the interaction between the microbiota and HPV, presenting the results of various studies that help identify potential biomarkers and therapeutic targets. However, there are also limitations. Most of the microbiota studies included in the review are designed to characterize the bacterial and cytokine profiles of the microbiota, making it difficult to conclude a causal relationship between the vaginal microbiome, HPV infection, and cervical precancerous lesions [11]. CST classification only partially explains the relationship between the microbiota and cervical conditions in women, as these microbiome profiles are highly dependent on bacterial species, which cannot be accurately profiled using commonly used sequencing technologies [49]. Additionally, studies have often used different methodologies, making it difficult to compare results. Some studies found that vaginal microbial diversity was not statistically significantly associated with the progression of cervical precancerous lesions, but this may have been due to differences in geographic locations, race of patients, and sample collection methods [84]. Many studies have limitations, such as relatively short follow-up times and small sample sizes, and the exclusion of patients with BV, who may have a more diverse vaginal microbiota [85].

Future studies should be conducted to determine the dynamics of microbiota changes and their impact on the persistence of HPV infection. Large-scale longitudinal cohort studies with regular cervical microbiota and HPV sampling are necessary to identify microbial markers associated with HPV clearance, persistence, and cancer progression [9,105]. Integrating 16S rRNA and shotgun approaches could provide a more comprehensive understanding of the microbiome’s role in cervical carcinogenesis [14]. Omics technologies (metagenomics, metabolomics, proteomics) are future tools necessary to assess the relationship between the cervical microbiota and cancer development [14,77,105]. Studying the role of the vaginal microbiota, together with the analysis of other external factors, could help to understand the pathogenesis of cervical diseases better and contribute to the development of more accurate diagnostics and more effective prevention and treatment strategies. It is important to integrate ethnic risk factors into cervical cancer screening and prevention programs to improve early detection and reduce disparities in outcomes [30].

## 5. Conclusions

This review provides evidence that the composition of the vaginal microbiota plays a dynamic and influential role in determining whether HPV infections resolve or progress to cervical precancerous conditions. Beyond reinforcing known associations, such as the protective role of *Lactobacillus crispatus* and the pathogenic potential of dysbiotic communities, this work highlights the need to integrate microbiome analysis into cervical disease risk assessment. These findings highlight the potential of microbiota profiling to improve risk assessment, facilitate personalized screening strategies, and inform the development of adjunctive therapies aimed at restoring microbial balance. By identifying key microbial patterns and clarifying their functional roles, this work provides a strong foundation for incorporating microbiome analysis into routine clinical practice. To advance toward clinical implementation, future studies should prioritize refining microbial classification methods and validating the outcomes of interventions across diverse populations.

## Data Availability

Data sharing is not applicable to this article.

## Appendix A

**Table A1:** Overview of studies analyzing the association between vaginal microbiota composition, HPV infection, and the progression of cervical intraepithelial lesions and cancer.

Authors, Year	Participants	Sample Types	Analysis Method	Healthy Patients	CIN1 or LSIL	CIN2/3 or HSIL	Cervical Cancer	Key Findings
Norenhag et al., 2024 [47]	354	Cervical and vaginal samples	Shotgun metagenomics sequencing	*L. crispatus* *L. iners*	*L. iners*, *G. vaginalis*, *Peptoniphylus* *lacrimalis*, *Megasphaera* spp.	*Anaerobic bacteria*, *Gardnerella* *vaginalis*, *Prevotella* spp., *Alloscardovia* *omnicolens*	No data	The CIN group was characterized by microbial diversity, more genes involved in nucleotide biosynthesis, and peptidoglycan biosynthesis. In the control group, the abundance of genes related to amino acid biosynthesis (L-lysine) and sugar degradation was higher.
Zheng et al., 2023 [79]	341	Cervical cells, tissue and vaginal secretions	Shotgun metagenomic sequencing	*L. crispatus* *L. iners*	*Gardnerella vaginalis*, *L. iners*	*Gardnerella vaginalis*, *L. iners*, *S. aureus*	*Staphylococcus aureus*, *Phocaeicolavulgatus*, *Salmonella enterica*, *G. vaginalis*, *Bacteroides fragilis*	Dysbiosis of the cervical and vaginal microbiota may lead to the enrichment of CpG motifs, which is associated with increased TLR9 expression and may promote the progression of cervical lesions.
Liu et al., 2022 [20]	115	Cervical cells and vaginal secretions	Shotgun metagenomic sequencing	*Lactobacillus* spp.	*Gardnerella vaginalis*, *Prevotella bivia*	*G. vaginalis*, *Prevotella* spp., *Anaerobic bacteria*	*Anaerobic bacteria* CST IV	As cervical lesions progressed, *Lactobacillus abundance* decreased, anaerobic and aerobic bacteria increased, HPV prevalence and *E6/E7* oncogene expression were observed.
Kwon et al., 2019 [80]	47	Cervical cells and vaginal secretions	Shotgun metagenomic sequencing	*Pseudoalteromonas*, *Psychrobacter*	No data	*Lactobacillus*, *Staphylococcus*, *Candidatus Endolissoclinum*	*Alkaliphilus*, *Pseudothermotoga*, *Wolbachia*	As CIN progresses, peptidoglycan synthesis processes increase, dioxin degradation and 4-oxalocrotonate tautomerase activities decrease.
So et al., 2020 [60]	50	Cervicovaginal swab specimens	16S rRNA V3–V4 region	*Lactobacillus* spp.	*L. iners*, *Sneathia sanguinegens*, *Gardnerella vaginalis*, *Prevotella timonensis*, *Prevotella buccalis*	*L. iners*, *G. vaginalis*, *Sneathia sanguinegens*, *Atopobium vaginae*, *Porphyromonas somerae*, *Prevotella disiens*	*Fusobacterium* spp., *Porphyromonas* spp. *Prevotella timonensis*, *Escherichia fergusonii*, *Haemophilus*	G. vaginalis is associated with persistent HPV infection and a high risk of developing CIN2/3 and cervical cancer.
Teka et al., 2023 [83]	120	Cervical cells collected using different brushes	16S rRNA V4 region	*Lactobacillus* spp.	*L. iners*	*L. iners*	*Porphyromonas somerae*, *Prevotella timonensis*, *Porphyromonas asaccharolytica*, *Bacteroides*, *Anaerococcus*	The diversity, composition and relative abundance of the microbiota differ between women with cervical cancer, women with dysplasia, and healthy women.
Yang et al., 2024 [106]	26	Cervical smear	16S rRNA V3–V4 region	*Lactobacillus* spp.	*L. iners.* *Atopobium*, *Prevotella*, *Bacteroides*	*Sneathia* *Firmicutes* *Proteobacteria*	No data	HPV infection affects metabolic signals, inducing changes in glycolysis, lactate accumulation, and lipid metabolism. Specific metabolites have been identified to distinguish healthy patients from patients with persistent HR-HPV infection.
Wu et al., 2021 [87]	94	Cervical smear	16S rRNA V4 region	*Lactobacillus* spp.	*Lactobacillus*, *Xanthobacter*, *Thermus*, *Flavisolibacter Sphingopyxis*, *Sediminibacterium*, *Geobacillus*	*Sneathia*, *Gardnerella*, *Megasphera*	*Porphyromonas*, *Campylobacter*, *Prevotella*	Identified potential markers of cervical cancer: *Porphyromonas* and *Campylobacter*. Functional metabolic pathways associated with Prevotella cause a negative effect, associated with *Lactobacillus*-positive status.
Molina et al., 2022 [49]	541	Cervical smear	16S rRNA V3–V4 region	*Lactobacillus* spp. *Lactobacillus* *acidophilus*	*L. iners*, *Gardnerella vaginalis.* *L. crispatus*	*Megasphaera genomosp 1 tipas*, *Atopobium vaginae*, *Dialister micraerophilus*, *Dialister micraerophilus*	*Megasphaera genomosp 1 type* *G. vaginalis*	The distribution of microbial communities in the microbiome reveals changes in microbial dynamics that may be associated with HR-HPV infection and cervical lesions.
Fan et al., 2024 [46]	125	Cervical smear	16S rRNA V4 region	*Phylum Firmicutes*, *Lactobacillus*, *Streptococcus*, *Varibaculum (biomarker)*	*Saccharopolyspora (biomarker)*, *Corynebacterium*	*Saccharopolyspora (biomarker)* *Prevotella*, *Megasphaera*, *Streptococcus*, *Sneathia*, *Dialister*, *Anaerococcus*, *Ureaplasma*, *Peptoniphilus*, *Mycoplasma*	*Proteobacteria*, *Corynebacterium*, *Coprococcus*, *Peptococcus*, *Ruminococcus (biomarkers)*, *Dialister*, *Peptoniphilus*	The microbiota has diagnostic value in distinguishing between healthy women, patients with CIN, and women with cervical lesions. In addition, microorganisms may be potential biomarkers for cervical cancer
Ma et al., 2023 [82]	160	Cervical smear	16S rRNA	*Lactobacillus* spp., *L. crispatus*, *Gardnerella*, *Prevotella*, *Fannyhessea vaginae*	*Lactobacillus* spp., *Sneathia*, *Dialister*, *Gardnerella*	*Lactobacillus* spp., *L. iners*, *Sneathia*, *Fastidiosipila Megasphaera*, *Prevotella*, *Bacteroides.*	*Prevotella*, *Bacteroides*, *Finegoldia*, *Gardanella*, *Streptococcus*, *Fannyhessea vaginae*, *Peptostreptococcus*, *Dialister*, *Veillonella*	A gradual decrease in *L. crispatus* and an increase in anaerobic bacteria are associated with the severity of cervical lesions. HPV 16 infection is associated with a greater diversity of vaginal microbiota and a decrease in *Lactobacillus*. *L. crispatus* and *L. iners* can be considered as biomarkers.
Liu et al., 2023 [85]	692	Vaginal discharge	16S rRNA V4 region	*Lactobacillus* spp.	*Ercella*, *Bacillus*, *B Lautia*, *Terrisporobacter*, *Sporobacter*, *Romboutsia*	*Prevotella*, *Gardnerella*, *Sneathia*, *Megasphaera*, *Dialister*, *Ureaplasma*	No data	CIN lesions are characterized by a decrease in *Lactobacillus* and *Pseudomonas* and an increase in *Gardnerella*, *Prevotella* and *Dialister*. High-grade CIN may inhibit the phosphotransferase system, transcription factors, fructose and mannose metabolism, aminosugar and nucleotide metabolism, and galactose metabolism.
Wu et al., 2020 [84]	69	Cervical smear	16S rRNA V3–V4 region	*Lactobacillus* spp.,	*Lactobacillus*, *Gardnerella*, *Atopobium*, *Sneathia*, *Streptococcus*	Delftia (*biomarker*), *Gardnerella*, *Prevotella*, *Atopobium*, *Sneathia*, *Streptococcus*	No data	The vaginal microbiota is directly or indirectly associated with the progression of CIN.

CIN, vaginal community state type; LSIL, low-grade squamous intraepithelial lesions; HSIL, high-grade squamous intraepithelial lesions; CC, cervical cancer, CpG, cytosine–phosphate–guanine; TLR9, toll-like receptor 9, 16S V3–V4, a gene region important for bacterial identification; Shotgun, analysis of total microbial DNA.
